# Faecal Microbiota Transplantation Modulates Morphine Addictive‐Like Behaviours Through Hippocampal Metaplasticity

**DOI:** 10.1111/adb.70034

**Published:** 2025-04-16

**Authors:** Negin Saeedi, Fereshteh Pourabdolhossein, Masoud Dadashi, Ali Jaafari Suha, Mahyar Janahmadi, Gila Behzadi, Narges Hosseinmardi

**Affiliations:** ^1^ Department of Physiology, School of Medicine Shahid Beheshti University of Medical Sciences Tehran Iran; ^2^ Department of Physiology, School of Medicine Babol University of Medical Sciences Babol Iran; ^3^ Department of Microbiology, School of Medicine Alborz University of Medical Sciences Karaj Iran; ^4^ Neurophysiology Research Center, Department of Physiology, School of Medicine Shahid Beheshti University of Medical Sciences Tehran Iran

**Keywords:** dependence, gut microbiota, gut–brain axis, hippocampus, opioid addiction, synaptic plasticity

## Abstract

The microbiota–gut–brain axis has been implicated in the pathology of substance use disorders (SUDs). In light of the brain's capability to reorganize itself in response to intrinsic and extrinsic stimuli, opioid‐induced dysbiosis is likely to contribute to addictive behaviour through modulating neuroplasticity. In this study, a faecal microbiota transplantation (FMT) from a saline‐donor was performed on morphine‐treated rats to evaluate the effects of gut microbiota on morphine‐induced metaplasticity and addictive behaviours. Male Wistar rats were treated with subcutaneous injections of 10 mg/kg morphine sulphate every 12 h for 9 days in an effort to induce dependence. The withdrawal syndrome was precipitated by injecting naloxone (1.5 mg/kg, ip) after the final dose of morphine. The tolerance was induced by repeated morphine injections over a period of 7 days (10 mg/kg, once a day, ip). FMT was applied daily through gavage of processed faeces 1 week before and during the morphine treatment. Field potential recordings (i.e., fEPSP) were carried out to assess short‐term and long‐term synaptic plasticity in the CA1 area of the hippocampus following Schaffer‐collateral stimulation. Animals subjected to FMT exhibited significant reductions in naloxone‐precipitated withdrawal syndrome (one‐way ANOVA, *p* < 0.05). Tolerance to the analgesic effects of morphine was not affected by FMT (two‐way ANOVA, *p* > 0.05). Following high‐frequency stimulation (HFS) to induce long‐term potentiation (LTP), a greater fEPSP slope was observed in morphine‐treated animals (unpaired *t* test, *p* < 0.05). FMT from saline‐donor rats diminished morphine‐induced augmented LTP (unpaired *t* test, *p* < 0.05). These results highlighted the alleviating effects of FMT from saline‐donors on morphine‐induced metaplasticity and dependence potentially by modulating the dysbiosis of gut microbiota.

## Graphical abstract

Behavioral features of addiction, such as dependence and tolerance to the analgesic effects of morphine and changes in synaptic plasticity in the hippocampus, can be influenced by alterations in the gut microbiota population and the fecal microbiota transplantation process.

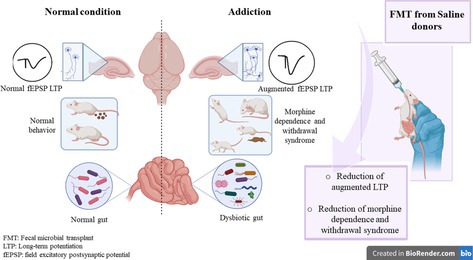

## 1 Introduction

The gut–brain axis is an integrated physiological concept involving neural, immune and endocrine channels between the gastrointestinal and central nervous systems [1]. As the brain and the intestine are physically separated, it seems harder to demonstrate a direct interaction between them [2]. In this sense, a bidirectional communication system with a shared language is essential for transmitting information from the gut to the brain. This communication is shaped by the trillions of microorganisms present in the gut, collectively called the gut microbiota [3]. The innovative hypothesis of the impact of the gut microbiota on the brain was further developed in 2011, which later elucidated the concept of the ‘microbiota–gut–brain axis’ [4]. Recently, research based on the microbiota–gut–brain axis theory indicated that gut microbiota might be influential in brain function and neuropathology [5], and a wide range of central nervous system diseases can benefit from its use as a biomarker [6].

Gut microbiota are the most intricate and diversified microecosystems in the human body [7], and their homeostatic balance is imperative for achieving good health [8]. A dysbiosis is characterized by an imbalance of the gut microbiota or its functions [9]. A perturbation in the gut microbiota has been confirmed to cause significant health problems and is linked to various pathological conditions, most notably neurodegenerative and neuropsychiatric disorders, as well as addiction [10, 11, 12].

Globally, opioid addiction is a chronic public health concern [13, 14], in which addicted individuals take substances despite their knowledge of their potentially hazardous effects [15]. It has been confirmed recently that drugs of abuse modify the gut microbiota composition [16]. A number of studies have proven that morphine treatment reduces Gram‐negative *Bacteroidetes* and increases Gram‐positive *Firmicutes*, containing potentially pathogenic bacteria of the *Enterococcaceae* and *Staphylococcaceae* families [17]. However, whether opioid‐induced dysbiosis make alterations in the neurobehavioral disorders is still under debate.

It is attractive to hypothesize that drugs of abuse cause long‐term changes on behaviour by altering synaptic function and plasticity in brain circuits [18]. Neurons are capable of modifying the strength of their connections due to synaptic plasticity [19]. The concept of metaplasticity refers to a form of higher order plasticity that is not exclusively about a change in synaptic transmission effectiveness, but rather about an alteration in the threshold for triggering plasticity. Considered the gatekeeper of synaptic plasticity, metaplasticity is a key determinant of drug‐induced neuroadaptations in the brain [20]. Research has indicated that chronic opioid use activates reward pathways in the brain. This leading to enduring modifications in brain plasticity and addictive drug behaviour, accompanied by major side effects such as dependence, relapse and tolerance [21, 22, 23]. As one of the limbic system's structures, the hippocampus plays a crucial role in learning, memory and mood regulation. In addition to being critical to long‐term memory formation, this structure plays a key role in the development of addictive memory [24]. The hippocampus is exceptionally susceptible to internal and external stimulation, especially gut dysbiosis [25].

Various factors, including neurogenesis, activation of microglia and hippocampal plasticity, are modulated by the microbiota [26, 27]. Studies on germ‐free (GF) mice provide the most compelling evidence that the gut microbiota plays a critical role in brain development and function [28]. Therefore, it would be valuable to investigate whether the microbiota modulate addictive‐like behaviours through the neural mechanism involved in addiction such as metaplasticity.

Manipulation of the gut microbiota through faecal microbiota transplantation methods can regulate behavioural properties. As an illustration, transplantation of faecal microbiota from alcoholic individuals and mice into healthy mice resulted in the development of symptoms associated with depression and anxiety induced by withdrawal [29, 30]. Faecal microbiota transplantation is currently being studied in rodents with opioid‐induced dysbiosis as a strategy to recover microbial homeostasis [1]. In this study, FMT was applied to evaluate the contribution of morphine‐induced microbiota modulation on addictive‐like behaviours justified by hippocampal metaplasticity.

## 2 Materials and Methods

### 2.1 Animals

From our own breeding, we obtained 80 male Wistar rats weighing 180–200 g. The cage bedding was replaced every other day for all rats throughout the experiment. All animals were kept on a 12‐h light/dark cycle (lights on from 7 am to 7 pm). A temperature‐ (22°C ± 1°C) and humidity‐controlled facility was maintained with food and water readily available at all times.

The behavioural and electrophysiological tests were all conducted during the light phase. Carbon dioxide chambers were utilized to sacrifice the rats at the end of the tests. All procedures were conducted in accordance with the National Institutes of Health Guide for the Care and Use of Laboratory Animals and were approved by Medical Sciences Ethics Committee guidelines of Shahid Beheshti University of Medical Sciences (IR.SBMU.MSP.REC.1400.243).

### 2.2 Drugs

Morphine sulphate (Temad, Iran), naloxone hydrochloride (Sigma Aldrich, St. Louis, MO), and urethane (Sigma, USA) were administered in this study. PBS or 0.9% sterile saline was used to dissolve the drugs.

### 2.3 Behavioural Manifestations of Addiction

#### 2.3.1 Morphine Dependence and Naloxone‐Precipitated Withdrawal

**FIGURE 1:**
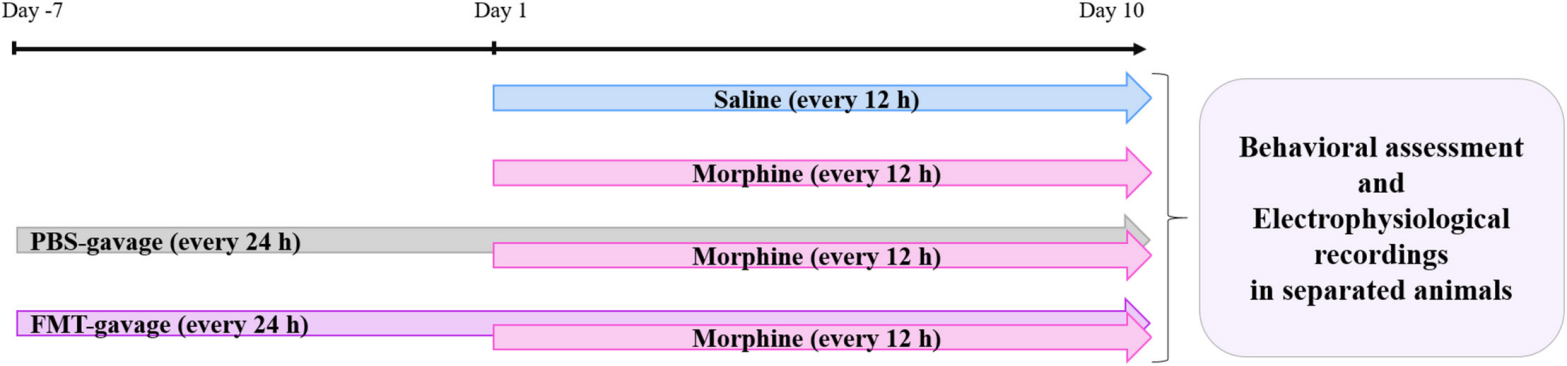
The schematic of study timeline indicating the timing of interventions, behavioural and electrophysiological assessments. FMT: faecal microbiota transplantation.

Morphine dependence was induced by repeated subcutaneous injections of 10 mg/kg morphine sulphate every 12 h (7 am and 7 pm) for nine consecutive days. Saline injection was conducted in the same manner as a control group. In order to ensure that the animals become dependent and to induce withdrawal syndrome, the animals received a final dose of morphine on 10^th^ day, and 2 h later, single dose of naloxone (1.5 mg/kg, ip) was injected to precipitate withdrawal syndrome. After naloxone injection, the rat was placed inside a rectangular cube with a length of 30 cm and a height of 50 cm on the basis of our previous study [31], and a blinded observer documented the number of withdrawal symptoms for 25 min. The Plexiglas chamber was disinfected before placement of each rat, while no other animals were allowed to enter the chamber during the behavioural assessments. The experimental procedure is shown in Figure 1. The definition of withdrawal signs is demonstrated in Table 1 [32].

**TABLE 1:** Definitions of morphine withdrawal signs used for behavioural ratings.

	Signs	Definitions
1	Activity	Crossing of a quadrant mark (A 15 × 15 cm^2^)
2	Chewing	Mastication of bedding of faecal material or chewing without any matter on the mouth
3	Defecation	Movement of faeces out the anus
4	Diarrhoea	Watery faeces
5	Freezing	Immobility for > 10 s
6	Head tremor	Shaking of the head only, without shaking of body
7	Penis licking	Evidence of licking of the penis
8	Ptosis	Squinting of the eyes
9	Rearing	Lifting the forepaws off the ground
10	Scratching	Rubbing the back of neck or the top of head with both forepaws
11	Sniffing	Short audible inhalations, with elevation of the muzzle and movement of the nares and nasal vibrissae
12	Yawning	Opening the mouth and take a deep breath

#### 2.3.2 Development and Evaluation of Morphine‐Induced Tolerance in a Hot‐Plate Test

A hot‐plate test was conducted in separated animals to determine morphine's analgesic effect. Before any intervention, the surface temperature was held steady at 50°C ± 1°C, and 60‐s cut‐off time was considered in all experiments to protect from thermal damage.

For evaluating tolerance to opioid analgesic effects, an intraperitoneal injection of 10 mg/kg of morphine or 1 mL of saline was given once a day for seven consecutive days. Based on the protocol [33], 5 min before and 20 min after each morphine or saline injection, the animal was placed on a hot‐plate apparatus (Borj Sanat Azma, Iran). Whenever a rat was placed in the apparatus, its response latency was recorded in seconds (the first act of licking a hind paw or jumping out of the apparatus).

Morphine's antinociceptive effect was calculated in accordance with the following formula: percent of maximum possible effect (%MPE) = [(T1 − T0)/(T2 − T0)] × 100, where T0 is predrug latency (baseline thermal threshold), T1 is postdrug latency, and T2 is cut‐off time.

Computer‐connected video cameras were used to record all behavioural tests. One hour of habituation was provided for all rats in the behavioural testing room. The apparatus was properly disinfected and dried for each animal.

### 2.4 Faecal Microbiota Transplantation (FMT)

Faecal microbiota transplantation was performed in this study to explore the implications of dysbiosis in gut microbiota due to chronic morphine use on behavioural and synaptic plasticity modifications. This was accomplished using gavage of processed faeces from saline‐treated donor animals to morphine‐treated recipients. The procedure was performed to mitigate the dysbiosis caused by morphine. Male donor rats weighted approximately 200 g were divided into two groups: saline and morphine. Animals received either saline (1 mL) or morphine (10 mg/kg) subcutaneously every 12 h for 10 days. Two hours after the last morphine or saline injection (between 9 and 11 am) on the 10^th^ day, fresh faecal samples of donor rats were collected and pooled. Two hundred milligrams of faecal samples were immediately diluted with 1‐mL PBS. Afterward, the slurry was filtered through a 70‐μM cell strainer and centrifuged for 20 min at 6000 *g* to obtain the final suspension. A suspension containing 10^10^ CFU/mL was prepared and delivered instantly to the rats via oral gavage with a stainless‐steel bulb tilted gavage needle which is attached to a syringe in a volume of 2 mL [34]. To sustain the effects of FMT, the aforementioned procedure was carried out once a day for 17 consecutive days.

One week before treatment with morphine, saline‐treated donor animals' microbiota were gavaged every 24 h to recipient animals. A subcutaneous injection of morphine (10 mg/kg) every 12 h for 9 days was initiated on the eighth day of continual FMT gavage every 24 h outlined above. Two hours after the last injection of morphine on 10^th^ day, naloxone hydrochloride (1.5 mg/kg, ip) was given to induce withdrawal symptoms.

It is important to mention that in electrophysiological study FMT from saline‐treated donor rats was started 1 week prior to morphine injection and continued during the process. Rather than administering naloxone, electrophysiological evaluation was performed after 10 days of morphine treatment in separated animals. In the hot‐plate test, during a weeklong hot‐plate test, morphine injections were given daily along with FMT gavage.

### 2.5 Electrophysiological Assessment

#### 2.5.1 In Vivo Field Potential Recording

Rats were placed in a stereotaxic device (Stoelting Instruments, Wood Dale, IL, USA) under anaesthesia with urethane (1 g/kg, ip) to record electrophysiological activity. A recording electrode and a stimulation electrode were inserted in the stratum radiatum of CA1 and CA3 areas, respectively, in the following manner: CA1: AP = −2.8 mm posterior to the bregma, ML = ±1.8 mm lateral to the midline, DV = 2.5–3.5 mm from the skull surface and CA3 (Schaffer collateral [SC]–CA1 pathway): AP = −3 mm posterior to the bregma, ML = ±3.1 mm lateral to the midline, DV = 3–3.5 mm from the skull surface in accordance with Paxinos and Watson's atlas [35].

Through the use of an isolator (A365R, WPI, Sarasota, FL, USA), test stimuli (200 μs, 0.1 Hz, 50–500 μA) were applied to the CA3 area. Stable baseline recordings were considered when fEPSP slopes fluctuated less than 10% for at least 30 min. Upon attaining a steady‐state baseline response, input–output (I/O) curve was determined by applying incremental stimulus intensity (from 50 to 500 μA, 1‐min recording for every intensity) and a stimulus intensity that evoked a fEPSP slope about 50% of the maximum response was chosen as a test pulse. Afterwards, 20‐min baseline recordings was conducted.

To determine short‐term synaptic plasticity, all experimental groups were given paired‐pulse stimulation with an interpulse interval (IPI) of 20, 80, and 200 ms. The paired‐pulse ratio (PPR) is defined as the slope of fEPSP2/fEPSP1 and indicates either paired‐pulse facilitation or inhibition [36].

A high‐frequency stimulation (HFS) protocol of 200 Hz was applied to induce LTP after 20 min of promising baseline recordings. The 200‐Hz pattern used in this study consisted of 10 bursts of 15 stimuli, 200‐μs stimulus duration, with 10‐s interburst interval. The stimulus intensity that evoked a fEPSP slope of approximately 80% of the maximum response was chosen to apply HFS. After HFS, the fEPSP slope was recorded for 60 min by applying test pulse intensity. Synaptic potentiation was defined as a minimum of 20% slope change in this study.

The data were analysed offline at a sample rate of 10 kHz. Low and high pass filter was set on 1 Hz and 3 kHz, respectively.

#### 2.5.2 Histology

**FIGURE 2:**
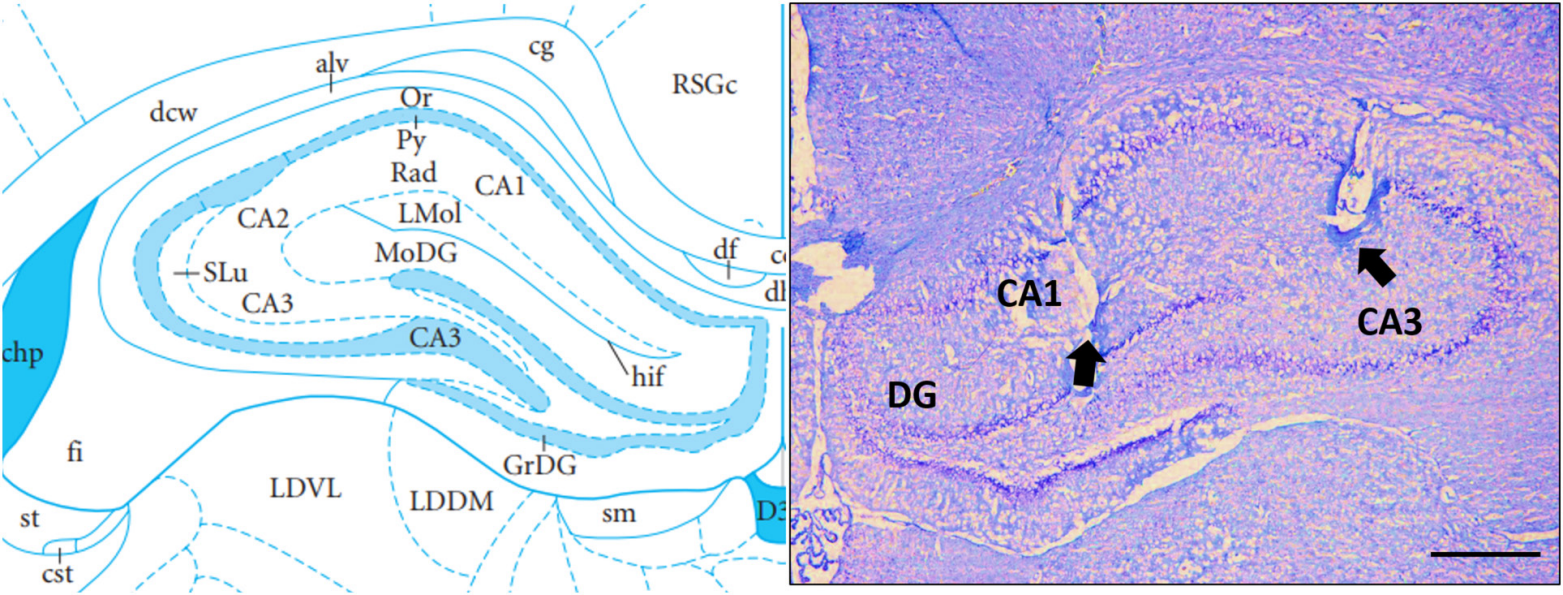
A cresyl violet–stained coronal brain section indicates the location of the stimulation and recording electrodes in CA1 and CA3 areas of the hippocampus. CA1: area CA1 of Ammon's horn; CA3: area CA3 of Ammon's horn; DG: dentate gyrus. Scale bar: 200 μm.

Upon completion of the electrophysiological experiment, animals were asleep on CO_2_. An electrode positioning evaluation was conducted by cutting 50‐μm coronal slices of the brains with a cryostat (Leica, Germany, CM1850). Figure 2 displays a cresyl violet–stained view of the recording and stimulating electrodes.

### 2.6 Data Analysis

In all experiments, the normality of the data was checked using the Kolmogorov–Smirnov test, and parametric or non‐parametric statistics were used accordingly. In order to investigate the parameters of withdrawal syndrome, group means were compared using unpaired Student's *t* test or Mann–Whitney *U* test. Two‐way and one‐way ANOVAs were used for analysis of %MPE and baseline responses in hot‐plate test, respectively. The electrophysiological data were analysed using two‐way ANOVA or paired/unpaired *t* test. Bonferroni's and Tukey's post hoc tests were used for multiple comparisons. GraphPad Prism 9.5.1 was used for all statistical analyses. In all experiments, significance threshold was set at *p* < 0.05.

## 3 Results

### 3.1 Withdrawal Symptoms in Morphine‐Treated Animals

**FIGURE 3:**
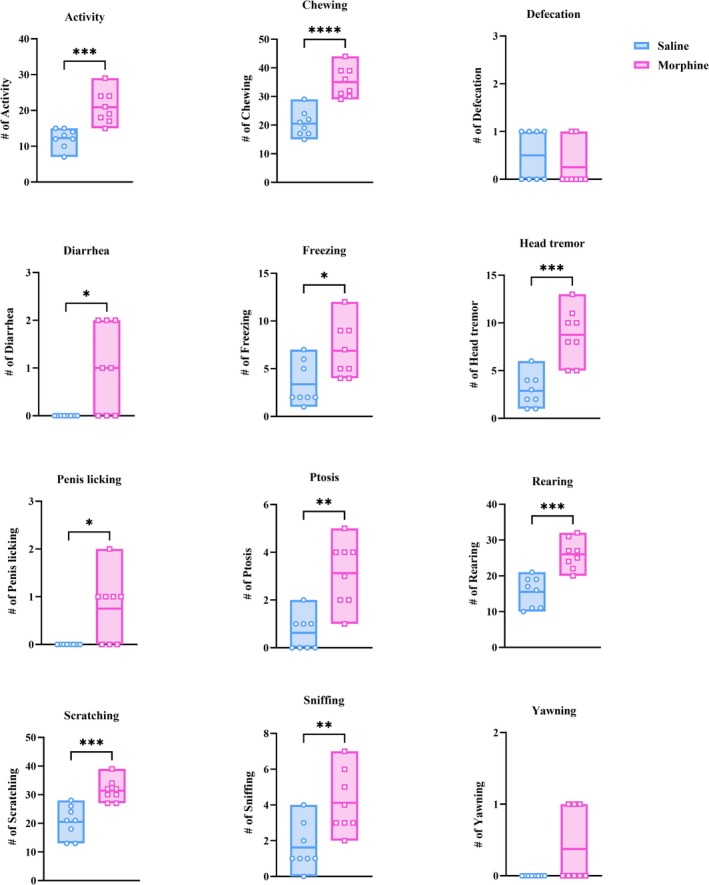
Behavioural manifestations of withdrawal syndrome in morphine‐dependent rats after naloxone injection. Symptoms of withdrawal‐like syndrome in animals administered saline (*n* = 8) are illustrated in comparison with the animals exposed to morphine (*n* = 8). All data are expressed as mean ± SEM. Unpaired *t* test or Mann–Whitney *U* test, *p < 0.05, **p < 0.01, ***p < 0.001, ****p < 0.0001.

Dependence was induced by injecting 10‐mg/kg morphine subcutaneously every 12 h for 9 days, followed by injection of 1.5 mg/kg naloxone on the 10^th^ day, after the last dose of morphine, to elicit withdrawal syndrome. There were significant differences between animals receiving morphine (*n* = 8) and saline (*n* = 8) with regard to withdrawal syndrome symptoms, likewise activity, chewing, head tremor, rearing and scratching (unpaired *t* test, *p* < 0.001); ptosis and sniffing (Mann–Whitney *U* test, *p* < 0.01); and diarrhoea, freezing and penis licking (Mann–Whitney *U* test, *p* < 0.05). Based on the protocol and the evident withdrawal symptoms, a rat model of morphine dependence has been developed (Figure 3).

**FIGURE 4:**
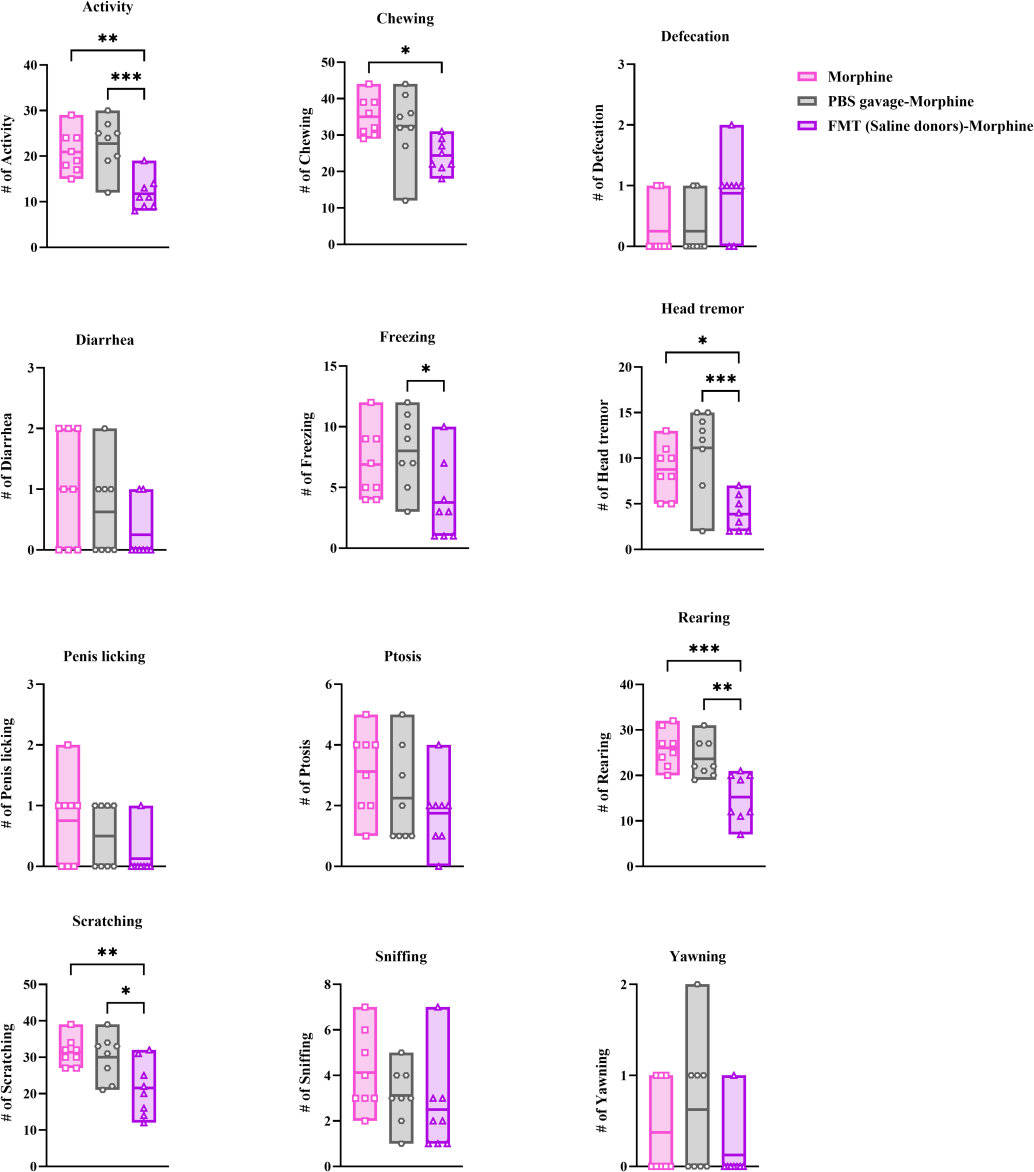
Behavioural manifestations of withdrawal syndrome in faecal microbiota transplanted morphine‐treated rats precipitated by naloxone. We explored withdrawal symptoms in animals with morphine injections and FMT gavage (*n* = 8) compared to those undergoing morphine injections and PBS gavage (*n* = 8). One‐way ANOVA followed by Tukey's post hoc test. *p < 0.05, **p < 0.01, ***p < 0.001.

In order to evaluate the role of dysbiosis in morphine dependence, an experiment was designed to find out whether repopulating the gut microbiota in morphine‐treated animals with the microbial community of saline‐donor animals (FMT) could modulate dependence. As compared to morphine group (*n* = 8), animals subjected to FMT from saline‐donor rats prior to morphine administration (*n* = 8) exhibited significant reductions in many withdrawal symptoms, such as rearing (*p* < 0.001), activity and scratching (*p* < 0.01), and chewing and head tremor (one‐way ANOVA, *p* < 0.05, Figure 4).

We observed that prevention of morphine effect on gut microbiota through FMT from saline‐donor animals could reduce naloxone‐precipitated withdrawal syndrome. We compared the morphine group with those receiving PBS, the solvent of the donor animals' microbiota suspension, to rule out the gavage procedure effect on behavioural symptoms. There were no significant differences between the morphine group and PBS‐gavaged morphine group (one‐way ANOVA, *p* > 0.05, Figure 4). Therefore, the reduction of behavioural symptoms in morphine dependence by FMT from saline‐donor rats could be due to prevention of presumed morphine‐induced dysbiosis.

Similar to morphine group, naloxone‐precipitated withdrawal symptoms, including activity and head tremor (*p* < 0.001), rearing (*p* < 0.01), freezing and scratching (*p* < 0.05) were lower in FMT (saline‐donor)‐morphine group compared to PBS‐gavaged‐morphine group (one‐way ANOVA, Figure 4).

### 3.2 Analgesic Effects of Morphine in a Hot‐Plate Test to Evaluate Tolerance

**FIGURE 5:**
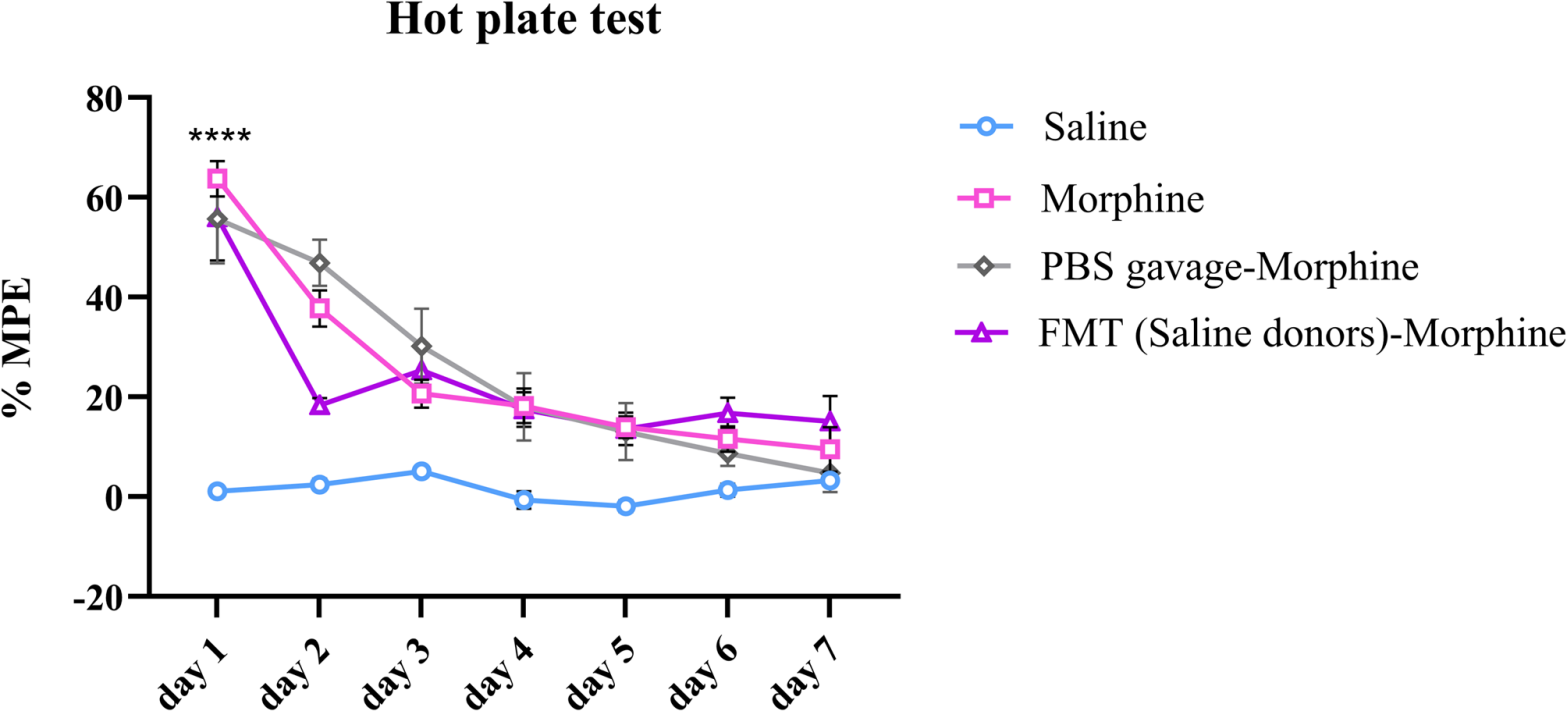
Alteration of the analgesic effect following repeated morphine administration in different groups during 7 days. All data are expressed as mean ± SEM. Repeated measure two‐way ANOVA followed by the Tukey's post hoc test. ****p < 0.0001.

Administration of morphine in all three morphine‐treated groups according to the previously mentioned protocol in hot‐plate test revealed analgesia compared to saline group (*F*(3, 20) = 35.4, two‐way ANOVA, *p* < 0.0001). On the first day in the morphine group, MPE was 63.8% compared to the saline group (MPE = 1.15%, *p* < 0.0001). Nevertheless, during 7 days, the percentage of MPE decreased gradually (*F*(6, 120) = 40.8, *p* < 0.0001), so that in the morphine group, there was a significant difference between Day 1 and other days, which indicates tolerance to the analgesic effects of morphine (from the percentage of MPE = 63.75% on the first day to 9.5% on the seventh day, *p* < 0.01, Figure 5).

In the PBS‐gavaged morphine group, the analgesic effects of morphine were observed on the first day (MPE = 55.7%). Similar to the morphine group, a significant difference was found in MPE between Day 2 and Day 6 (from 46.83% to 8.71%, *p* = 0.0126) and Day 2 and Day 7 (from 46.83% to 4.78%, *p* = 0.0207, Figure 5).

Also, in the FMT (saline‐donors)‐morphine group, compared to the saline group, we observed analgesic effect on the first day (MPE = 56%, two‐way ANOVA, *p* = 0.0049). In this group, there was a significant difference between Day 1 and Day 2 (MPE = 18.35%, *p* = 0.0453) and Day 4 (MPE = 17.51%, *p* = 0.0182, Figure 5). Here, like morphine group, in the FMT (saline‐donors)‐morphine group, a reduction in MPE and tolerance to the analgesic effects of morphine was observed.

**FIGURE 6:**
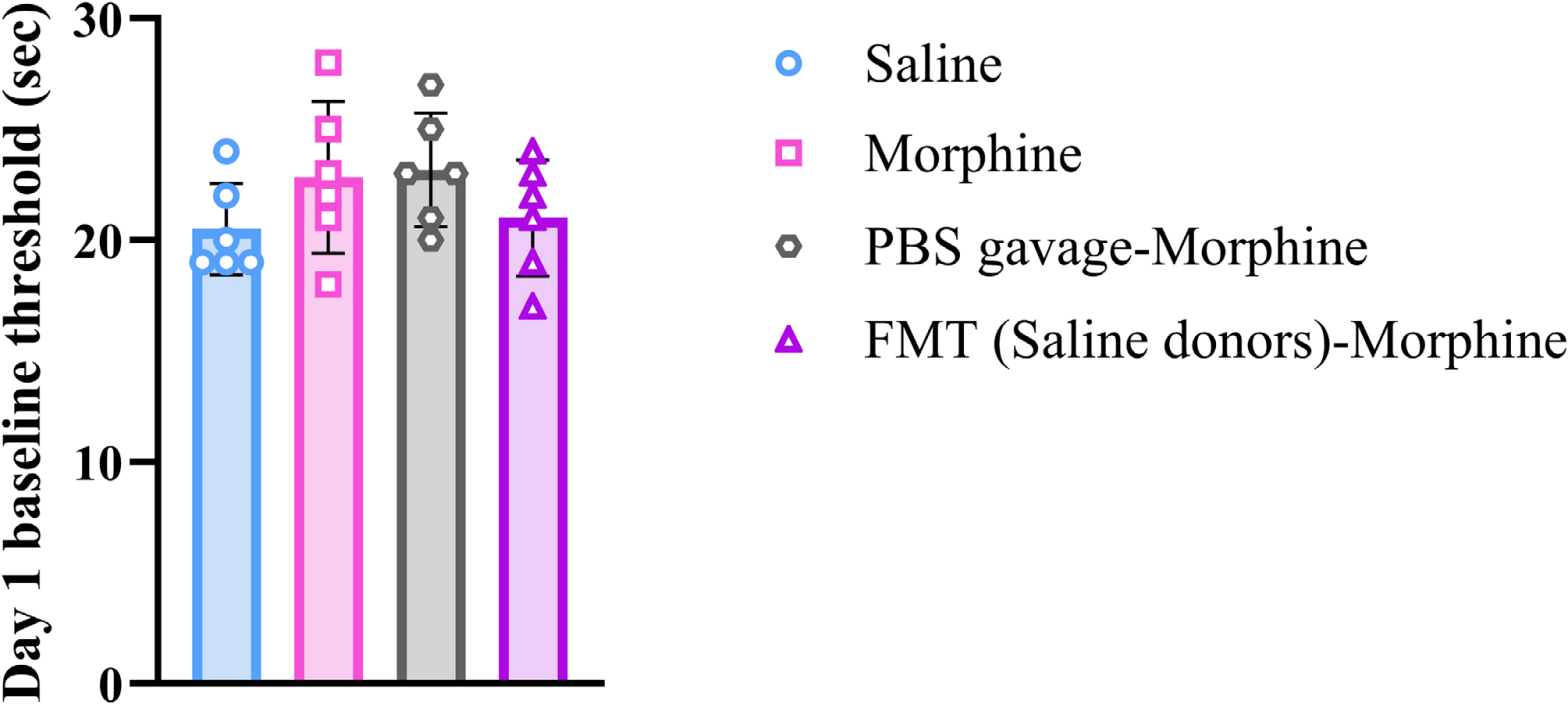
Effects of saline and morphine administration and FMT from saline‐donor animals to morphine‐treated rats and PBS gavage in morphine‐treated rats on Day 1 baseline threshold. One‐way ANOVA followed by the Tukey's post hoc test.

Because FMT was started before morphine administration, baseline responses were assessed for the first day. One‐way ANOVA showed no significant difference between all groups (*F*(3, 20) = 1.43, *p* = 0.2650, Figure 6).

### 3.3 Field Potential Recordings

#### 3.3.1 Baseline Synaptic Responses in CA1 Area of the Hippocampus

A single pulse was applied in a frequency of 0.1 Hz to stimulate the SC inputs to CA1 neurons. One fEPSP was elicited for each pulse in the stratum radiatum. For each given stimulus intensity, the responses were averaged over six traces. I/O curves were derived based on the slopes of the evoked field potentials for incremental stimulus intensity. Intensity threshold (*T*) is described as that which elicits measurable responses at the lowest intensity. Table 2 presents the average stimulus intensity (μA) required to elicit a baseline synaptic response in each animal. Incremental intensities were applied at five intensities ranging from *T* to 5*T* μA. Neither group revealed significant differences in stimulus intensity (*F*(3, 12) = 6.154, *p* > 0.05, two‐way ANOVA, Bonferroni post‐test, Table 2). Experimental groups did not vary significantly in the stimulus intensity required to provoke the maximum response (*I*_max_) and 50% of the maximal response (*I*_Test Pulse_) (*F*(3, 43) = 0.5895, *p* = 0.6252, two‐way ANOVA, Bonferroni post‐test, Table 3).

**TABLE 2:** Indication of the mean stimulus intensity of different experimental groups.

Groups	Stimulus intensity				
	1*T* (μA)	2*T* (μA)	3*T* (μA)	4*T* (μA)	5*T* (μA)
Saline	57.28 ± 5.39	79.3 ± 8.61	110.41 ± 19.83	159.3 ± 12.6	195.27 ± 18.11
Morphine	78.3 ± 4.14	111.74 ± 10.5	134.07 ± 11.9	171.64 ± 8.41	199.48 ± 12.05
PBS gavaged morphine	69.42 ± 6.78	107.55 ± 9.04	139.47 ± 6.17	175.3 ± 10.38	187.6 ± 9.12
FMT (saline donors) morphine	73.57 ± 8.26	92.8 ± 10.23	125.75 ± 10.3	178.2 ± 17.3	201.16 ± 13.24

*Note:* The threshold intensity (1*T*) is the lowest intensity that elicits a measurable response, while 2*T*–5*T* represent other intensities tested. An analysis of variance was performed on the data in order to compare multiple groups (two‐way ANOVA). The level of significance for differences was set at *p* < 0.05.

**TABLE 3:** The field response was captured in the rats' stratum radiatum of the CA1 region, which is evoked by a single shock in Schaffer collaterals.

Groups	*I*_max_ ^a^ (μA)	Max fEPSP slope (mV/mS)	*I*_Test Pulse_ ^b^ (μA)	Test fEPSP slope (mV/mS)
Saline	195.27 ± 18.11	0.51 ± 0.05	99.56 ± 4.89	0.13 ± 0.02
Morphine	199.48 ± 12.05	0.7 ± 0.04	120.2 ± 4.36	0.21 ± 0.02
PBS‐gavaged morphine	187.6 ± 9.12	0.56 ± 0.04	127.47 ± 6.9	0.18 ± 0.02
FMT (saline donors) morphine	201.16 ± 13.24	0.45 ± 0.05	100.13 ± 7.72	0.10 ± 0.01

*Note:* The values are expressed as Mean ± SEM. Baseline parameters did not differ significantly between the groups. Two‐way analysis of variance was used to compare data from multiple groups. *p* < 0.05 was statistically significant for differences.

^a^ *I*_max_ is the stimulus intensity evoking the maximum response (max fEPSP slope).

^b^ *I*_Test Pulse_ is the stimulus intensity that yielded 50%–60% of the fEPSP slope (test fEPSP slope).

**FIGURE 7:**
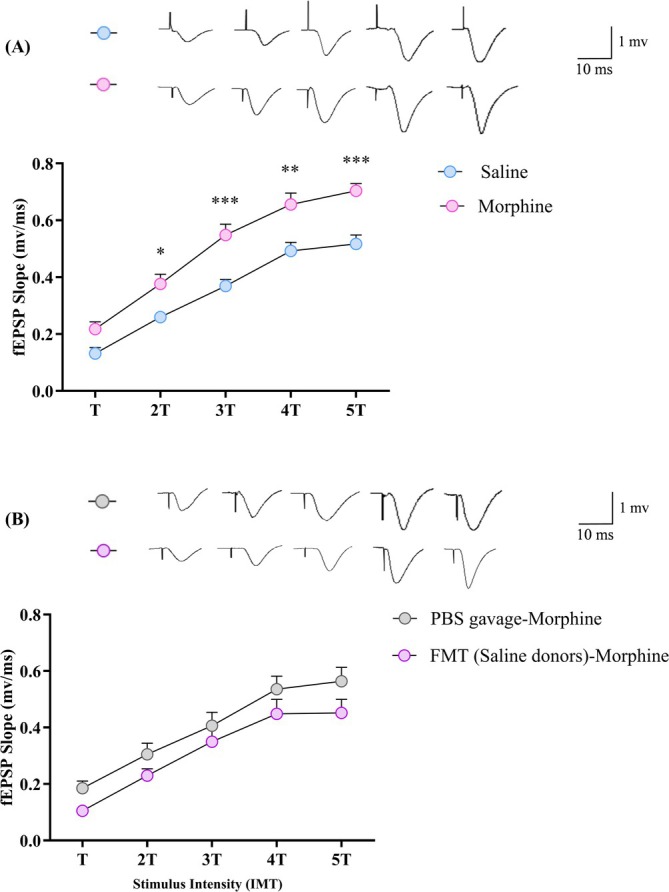
Input–output (I/O) curve in response to incremental stimulus intensity. (A) Saline and morphine‐treated rats and (B) PBS‐gavaged and FMT‐gavaged morphine‐treated rats were compared for synaptic I/O relations between Schaffer‐collateral and CA1 pyramidal cells. IMT: integer multiples of threshold intensity. (Two‐way ANOVA, *p < 0.05, **p < 0.01, ***p < 0.001). The insets provide views of the sample fEPSP traces obtained under different levels of stimulation with a calibration bar of 1 mV, 10 ms.

I/O curve was plotted for each experimental group (Figure 7). Treatment with morphine significantly modified the mean value of fEPSP slope (*F*(1, 10) = 16.31, *p* = 0.0024, two‐way ANOVA, Bonferroni post‐test). Considering 2*T*, 3*T*, 4*T* and 5*T* stimulus intensities, morphine‐treated rats (*n* = 6) indicated significantly greater slopes of fEPSP than saline‐treated rats (*n* = 6) (two‐way ANOVA, Bonferroni post‐test, *p* < 0.05, *p* < 0.001, *p* < 0.0001, Figure 7A). These data imply that the baseline synaptic response can be influenced by morphine treatment. However, daily faecal microbiota transplantation from saline‐treated donors to morphine‐treated animals did not change baseline synaptic responses (*F*(1, 10) = 2.42, *p* = 0.151, two‐way ANOVA, Bonferroni post‐test, Figure 7B).

As the behavioural study found no significant difference between the PBS‐gavaged morphine and morphine group, these groups were not compared regarding the electrophysiological parameters.

#### 3.3.2 Short‐Term Synaptic Plasticity in Response to Paired‐Pulse Stimulations

**FIGURE 8:**
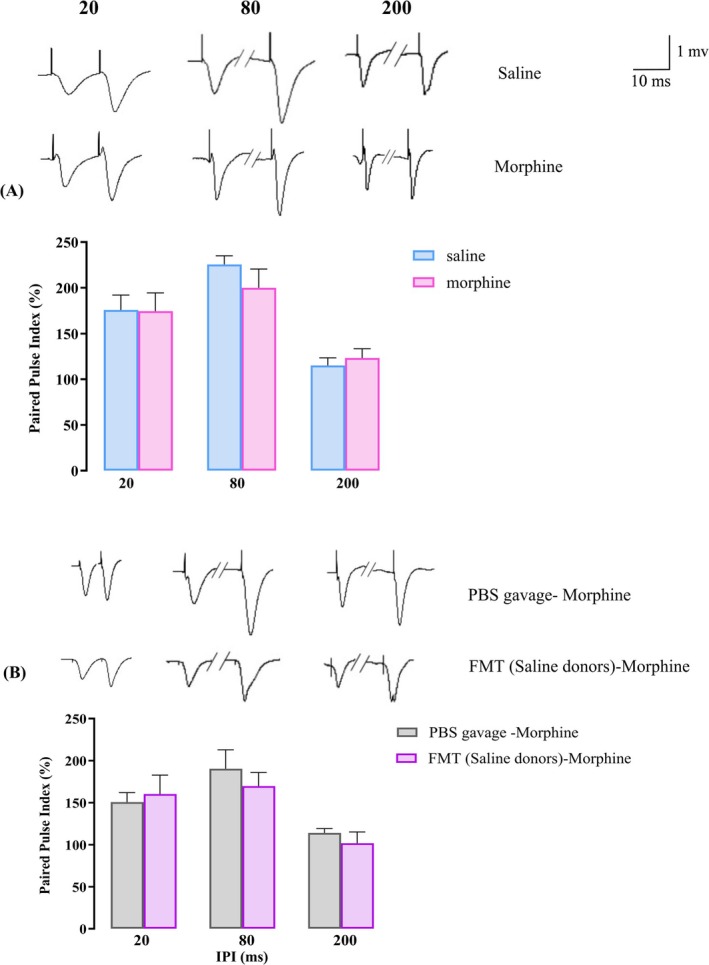
Short‐term synaptic plasticity in response to paired‐pulse stimulations. The paired‐pulse ratio of the fEPSP slope was measured at 20‐, 80‐ and 200‐ms IPI in (A) the saline and morphine‐treated rats and (B) PBS‐gavaged and FMT‐gavaged morphine‐treated rats. The bar graph represents mean ± SEM. Two‐way ANOVA. Calibration bar: 1 mV, 10 ms.

If two excitatory stimuli occur within a short space of time of each other, the second postsynaptic response, under normal conditions, will be enhanced [38]. This short‐term facilitation is mainly a result of presynaptic mechanisms leading to an increase in the amount of neurotransmitter release [39]. We tested short‐term synaptic plasticity using paired‐pulse stimulus with IPI of 20, 80, and 200 ms to assess the effects of chronic morphine administration on hippocampal circuit level. Morphine exposure did not change the PPR (*F*(1, 10) = 0.2024, *p* = 0.662, two‐way ANOVA, Figure 8A). Additionally, FMT from saline‐treated donor animals did not alter 20‐, 80‐ and 200‐ms IPI responses as compared to the PBS‐gavaged morphine group (*F*(1, 10) = 0.159, *p* = 0.7, two‐way ANOVA, Figure 8B).

#### 3.3.3 Long‐Term Synaptic Plasticity in Response to HFSs

**FIGURE 9:**
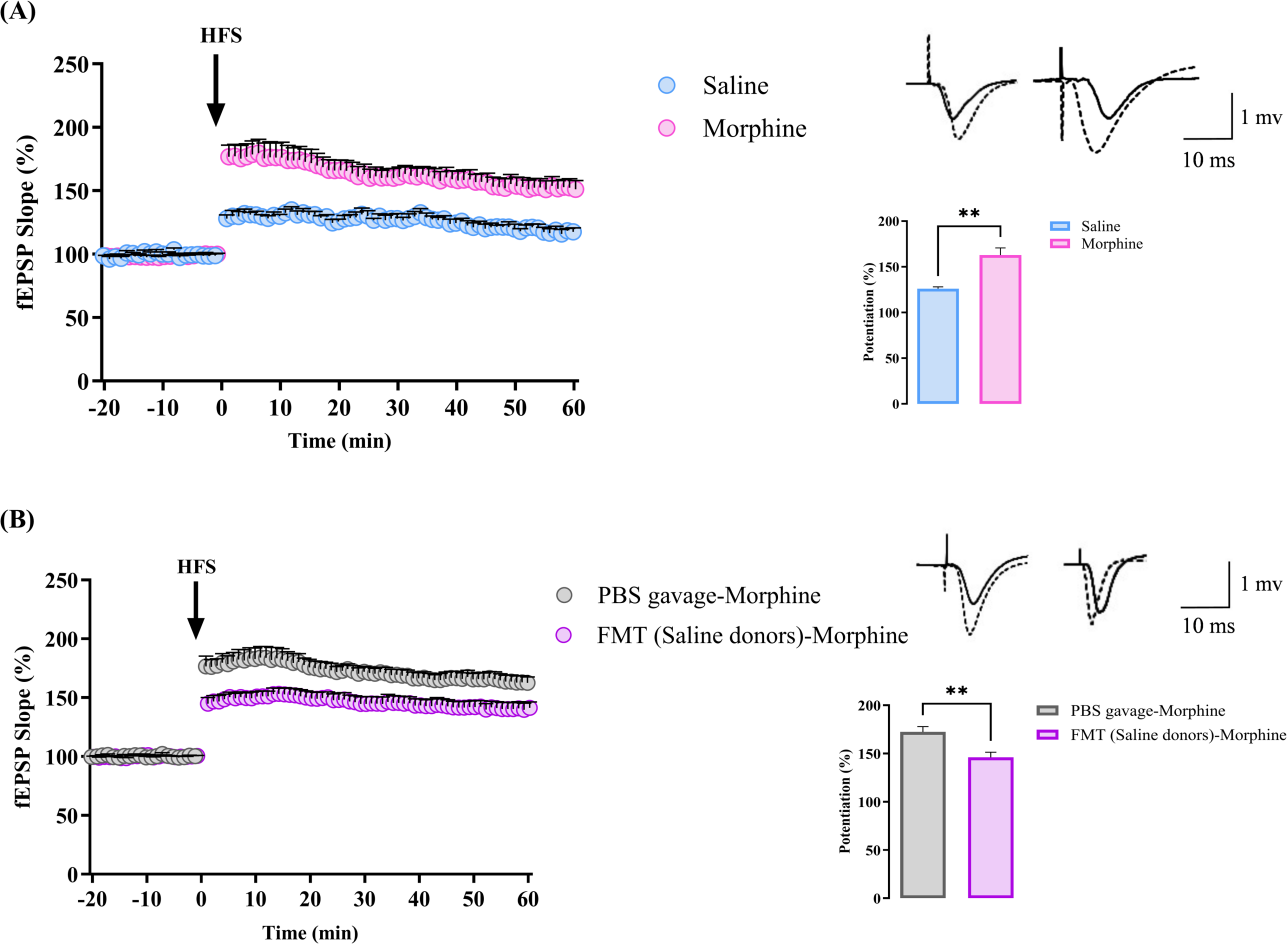
Long‐term synaptic plasticity in response to high‐frequency stimulations (HFSs). (A) fEPSP slope (%) versus time before and after HFS in saline‐ and morphine‐treated rats. (B) fEPSP slope (%) in PBS‐gavaged and FMT‐gavaged morphine‐treated rats. Insets indicating an average of the fEPSP slope change (%) during 60 min post‐HFS in the bar graphs. Bar graph represents mean ± SEM. **p < 0.01, unpaired *t* test. Sample traces before (solid line) and after (dashed line) HFS are displayed. Calibration bar: 1 mV, 10 ms.

The levels of synaptic plasticity in the hippocampal CA1 region are altered by several drugs of abuse. Due to this, an examination of LTP in this brain region would be crucial to better comprehend addiction mechanisms [36]. Using HFS, we assessed morphine's impact on synaptic plasticity in the SC–CA1 pathway following baseline recordings. fEPSP slopes were normalized after applying HFS based on baseline recordings for 20 min. The average response obtained for 60 min after HFS was compared to an average baseline response. As a result of our research, HFS increased the slope of fEPSP in both control (126.1% ± 2.02%, *n* = 6, *p* < 0.01, paired *t* test) and morphine (162.8% ± 7.85%, *n* = 6, *p* < 0.01, paired *t* test) groups. However, morphine‐treated animals exhibited a significantly greater increase in fEPSP slope after HFS than saline‐treated animals (*p* < 0.01, unpaired *t* test, Figure 9A).

Our hypothesis proposed that dysbiosis could contribute to metaplasticity changes caused by drug abuse. We attempted to compensate for the changes in microbiota population following morphine consumption by using FMT. The augmentation of LTP caused by morphine was diminished by FMT from saline‐treated donor rats into morphine‐treated animals (PBS‐gavaged morphine: 172.279% ± 5.530%, *n* = 6, vs. FMT (saline donors) morphine: 146.03% ± 5.246%, *n* = 6, unpaired *t* test, *p* < 0.01, Figure 9B). As a result of these data, FMT from saline‐treated donors appears to diminish morphine‐induced augmentation of LTP in the SC–CA1 pathway.

## 4 Discussion

In the present study, we focused on the contribution of peripheral mechanisms, namely, microbiota–gut–brain axis, on morphine‐induced behavioural and electrophysiological characteristics of addiction. An injection of naloxone following 10 days of morphine administration caused withdrawal symptoms in morphine‐treated male rats in this study. It was interesting that FMT from saline‐donors alleviated withdrawal syndrome symptoms in morphine‐treated animals. In order to determine how morphine‐induced dysbiosis could affect behavioural characteristics of addiction, we examined metaplasticity in the hippocampus, which is one of the known mechanisms driving morphine side effects. The results here indicated that FMT from saline‐donors to animals receiving morphine diminished the morphine‐induced LTP augmentation.

A balanced gut microbiota is now widely recognized to be crucial to proper signalling of the microbiota–gut–brain axis, thereby affecting the host's overall health—from the gastrointestinal tract to the brain [9, 40]. Dysbiosis of the gut microbiota in humans [41, 42] and animals [37, 43] has been linked to opioid use from the perspective of drug addiction. It has been reported that opioid‐induced gut dysbiosis may contribute to the emergence of morphine's rewarding or reinforcing properties [3]. In conjunction with opioid side effects, physical dependence manifests itself as a need to continue taking drugs to avoid withdrawal symptoms [44]. It is probable that opioid withdrawal symptoms will lead to compulsive drug usage and short‐term relapses, hence increasing opioid addiction potential [45].

Researchers have recently demonstrated a strong association between chronic opioid use and altered gut microbiota composition in animals and in humans [3]. There seems to be a potential role for the gut microbiota in morphine dependence, including withdrawal symptoms. FMT is currently the most promising method for discovering the underlying relationship between gut microbiota and psychiatric disorders, including autism [46], schizophrenia [47], and substance use disorders [3].

Preclinical studies indicate that opioid treatment results in increased intestinal barrier permeability, which raises the likelihood of bacteria transferring into the bloodstream and other organs. Upon bacterial translocation from the gut, immune cells can release pro‐inflammatory cytokines, leading to neuroinflammation. The development of opioid dependence and withdrawal is clearly influenced by neuroinflammatory responses [3]. In this way, it appears that we have been able to prevent the occurrence of the above‐mentioned events by FMT from saline‐donor rats before morphine administration and the onset of neuroinflammation, which results in a reduction in withdrawal symptoms.

A number of studies have demonstrated that drugs of abuse induce aberrant changes in synaptic plasticity in a way that promotes substance abuse‐related behaviours [48]. Due to FMT's effect on behavioural changes, it was intriguing to know whether FMT influenced morphine‐induced hippocampal metaplasticity. It has been demonstrated that electrophysiological characteristics of conventional and GF mice showed the same basal synaptic excitability and reduced LTP in GF animals [4], in which gut microbiota were emphasized as important modulators of synaptic plasticity. In this study, we hypothesized a possible link between the gut microbiota and hippocampal synaptic plasticity modifications after opioid exposure. Here, we observed that morphine‐treated rats exhibited greater baseline response and synaptic potentiation when LTP was induced. It was interesting to note that faecal microbiota transplantations from saline‐donors in rats treated with morphine displayed a significant reduction in morphine‐induced increase in LTP. As previously stated, the inflammatory response to the consumption of morphine is well known. It has been demonstrated that the glutamatergic tone and excitatory neurotransmission are markedly enhanced upon neuroinflammatory responses [49]. Glutamate binds to α‐amino‐3‐hydroxy‐5‐methyl‐4‐isoxazolepropionic acid (AMPA) and NMDA (*N*‐methyl‐d‐aspartate) receptors and accumulates in the extracellular space owing to glutamate transporter 1 (GLT‐1) downregulation that further affect postsynaptic responses [50]. It is therefore not surprising that chronic morphine administration causes strengthening of LTP by increasing glutamate concentrations in the synaptic space.

Because morphine‐induced dysbiosis increases the levels of pro‐inflammatory cytokines, activated glial cells [51] in the brain can also orchestrate potent modulatory control over synaptic plasticity [48]. Considering the role of gut microbiota and glial cells in changing the concentration of some neurotransmitters and gliotransmitters in the synaptic space, the effect of opioids on synaptic plasticity could partly be due to its effect on the microbiota population. Also, studies have indicated that gut microbiota and TNFα are related. TNFα crucially regulate synaptic plasticity in a brain region–specific manner [48]. Hence, the effect of FMT on reducing aberrant plasticity caused by morphine might be due to maintaining the microbiota population and preventing TNFα and pro‐inflammatory cytokines from being released [52].

In another part of this study, short‐term synaptic plasticity was investigated. The evaluation of the results showed no change in the groups. Various mechanisms are involved in short‐term synaptic plasticity, including the role of GABAergic and glutamatergic circuits [36]. In spite of the fact that morphine can disturb the balance of gut microbiota, it is not clear how its pattern affects short‐term plasticity. In this regard, studies have documented normal presynaptic short‐term plasticity in adult GF mice compared to regularly raised mice [4].

Tolerance can occur due to changes in the nervous system caused by morphine. Therefore, another part of the study assessed how the microbiota affects the development of morphine tolerance. It has been stated that activation of microglia can be modulated through microbiota. For instance, activation of microglia results in the release of pro‐inflammatory cytokines and chemokines, which in turn induce hyperexcitability in nociceptive neurons, ultimately contributing to the development of morphine tolerance [1]. In this study, morphine injection led to tolerance to its analgesic effects on the second day. Studies on morphine tolerance have previously concentrated on the central nervous system. Morphine tolerance, however, cannot be fully explained by the available knowledge of the central nervous system. Given morphine's extensive effects on the gut, it has been speculated whether gut microbiota could participate in morphine tolerance [1]. Although studies on microbiota modification following FMT have been reported [28], the effectiveness of this modality in reducing tolerance to the analgesic effects of morphine is still debated [53]. For instance, FMT from placebo‐pelleted mice attenuated tolerance in chronic morphine‐treated mice. Nevertheless, FMT from morphine‐pelleted mice did not contrarily affect placebo‐pelleted mice [54]. It is noteworthy to clarify that these results conflict with a previous report, which mentioned the induction of tolerance in GF mice treated with FMT from chronic morphine‐treated animals [34]. On the other hand, it has been determined that FMT from normal mice did not significantly prevent the occurrence of tolerance to morphine analgesia after chronic morphine exposure [53]. Our findings in this study also indicated no changes in morphine tolerance to analgesic effects when FMT was performed compared to the morphine group. Recent studies demonstrated that analgesic tolerance to morphine disappeared when MOR was conditionally deleted from primary neurons, suggesting that peripheral primary neurons play a crucial role in morphine tolerance [55]. In view of these findings, it appears that the pattern of tolerance varies across specific days and different treatment regimens, and more evaluations in future investigations would be helpful.

The absence of gut microbiota population quantification is one of the study's shortcomings. As indicated previously, in numerous research, the assessment of the gut microbiota population has been undertaken using various approaches including whole genome sequencing. In this investigation, we were not able to perform this technique to accurately analyse the gut microbiota community. Future research is advised to assess alterations in the gut microbial population following both morphine administration and FMT, taking into account various morphine delivery schedules.

## 5 Conclusion

Overall, in this study, we have shown that modification of neuroplasticity following morphine consumption that corresponds with addictive behaviours could be driven by dysbiosis. Faecal microbiota transplantations from saline‐donors is applicable in this case to modulate the microbiota dysbiosis and stave off the manifestation of addiction‐related behaviours and withdrawal symptoms. Further research is needed to elucidate the underlying mechanisms.

## Ethics Statement

All procedures were conducted in accordance with the National Institutes of Health Guide for the Care and Use of Laboratory Animals and were approved by Medical Sciences Ethics Committee guidelines of Shahid Beheshti University of Medical Sciences (IR.SBMU.MSP.REC.1400.243).

## Conflicts of Interest

The authors declare no conflicts of interest.

## Data Availability

The data that support the findings of this study are available on request from the corresponding author. The data are not publicly available due to privacy or ethical restrictions.
